# Implicit bias in HIV testing based on indicator conditions in primary care: a population-based study in Catalonia, Spain, 2017 to 2021

**DOI:** 10.2807/1560-7917.ES.2025.30.24.2400585

**Published:** 2025-06-19

**Authors:** Cristina Agustí, Oriol Cunillera, Juanjo Mascort, Ricard Carrillo, Jordi Casabona

**Affiliations:** 1 Centre of epidemiological studies on sexually transmitted infections and AIDS of Catalunya (CEEISCAT), Department of Health, Government of Catalonia, Badalona, Spain; 2 Biomedical Research Center Network for Epidemiology and Public Health (CIBERESP), Instituto de Salud Carlos III, Madrid, Spain; 3 Germans Trias i Pujol Research Institute (IGTP), Campus Can Ruti, Badalona, Spain; 4 Metropolitan South Research Support Unit, Jordi Gol i Gurina Primary Health Care Research Institute Foundation (IDIAPJGol), l'Hospitalet de Llobregat, Barcelona, Spain; 5 Primary Care Centre Florida Sud, Catalan Health Institute, Hospitalet de Llobregat, Barcelona, Spain; 6 Department of Clinical Sciences, Faculty of Medicine, University of Barcelona, Barcelona, Spain; 7 Department of Genetics and Microbiology, Autonomous University of Barcelona, Badalona, Spain

**Keywords:** HIV testing, primary care, indicator conditions, implicit bias

## Abstract

**BACKGROUND:**

HIV testing guided by indicator condition (IC) is recommended by the World Health Organization to facilitate earlier diagnosis. However, it is unclear to what extent these guidelines are followed in routine primary care (PC).

**AIM:**

To estimate the prevalence and distribution of ICs in PC in Catalonia, Spain, identify factors associated with, and prevalence of, an HIV test being administered, or not, within 4 months of IC diagnosis and assess trends over time.

**METHODS:**

A population-based cross-sectional study was conducted using data from the Information System for the Development of Research in Primary Care, covering 5.8 million individuals in Catalonia. We identified IC episodes recorded from 1 January 2017 to 31 August 2021 among patients aged 16–65 years. For each IC episode, we assessed whether an HIV test was performed within 4 months.

**RESULTS:**

We identified 372,712 IC episodes; 84,694 (22.7%) led to an HIV test within 4 months. Testing was higher for: men (26.3% vs 19.4% for women); migrants (27.7% vs 21.3% for Spanish citizens); younger patients (29.2% for 16–30-year-olds vs 13.7% for those over 50 years). Testing rates were highest for episodes involving syphilis (68.4%), genital herpes (50.6%), chlamydia (48.2%) and gonorrhoea (43.1%). Factors associated with increased testing included male sex, higher socioeconomic deprivation area, presence of an acute sexually transmitted infection and multiple ICs.

**CONCLUSION:**

Substantial gaps remain in HIV testing based on ICs in PC in Catalonia. Targeted interventions are needed to improve adherence to IC-guided testing, enabling early HIV diagnosis and treatment.

**Key public health message**

**What did you want to address in this study and why?**

Despite World Health Organization and European Centre for Disease Prevention and Control recommendations, it is unclear how often HIV testing is offered after an indicator condition (IC) is diagnosed in routine primary care. We assessed the proportion of patients diagnosed with an IC and subsequently tested for HIV to identify factors associated with not being tested and gaps in HIV screening strategies.

**What have we learnt from this study?**

We found that only 22.7% of patients diagnosed with an HIV IC were tested for HIV within 4 months, with significant disparities based on sex, age, socioeconomic status and geographical location. The study also highlighted that certain ICs, such as sexually transmitted infections, were more likely to prompt HIV testing.

**What are the implications of your findings for public health?**

To improve early HIV detection, targeted interventions are needed to increase adherence to IC-guided testing protocols, particularly in under-tested populations such as women, patients aged 50 years or older and those living in rural areas. Training healthcare providers to recognise a broader range of ICs and addressing implicit biases can enhance HIV testing rates and reduce the incidence of undiagnosed HIV.

## Introduction

In 2022, 460 new HIV diagnoses were reported in Catalonia, Spain, corresponding to a rate of 5.9 cases per 100,000 population [1]. This rate is similar to other western European countries but slightly higher than the European Union average of 5.1 cases per 100,000 population [2]. Diagnostic delay (defined as CD4 < 350 cells/μL at time of diagnosis) was observed in 49.6% of cases, and 24.1% had a late diagnosis of advanced disease (CD4 < 200 cells/μL) [1]. Delayed diagnosis is associated with higher rates of morbidity and mortality, higher economic costs, a longer period of transmissibility and therefore a greater contribution to incidence [3-6].

A key strategy to facilitate timely diagnosis, endorsed by the World Health Organization (WHO) and European Centre for Disease Prevention and Control (ECDC), is HIV indicator condition (IC)-guided testing. It has been established that HIV IC-guided testing is a cost-effective strategy of discovering HIV in conditions with an undiagnosed HIV prevalence of more than 0.1% [7-9].

The WHO recommends provider-initiated HIV testing in conditions that could indicate HIV infection to facilitate timely diagnosis [10]. The HIV Indicator Diseases across Europe Study (HIDES) identified key ICs for HIV [7-9]. Indicator conditions for HIV were defined as conditions that (i) qualify as AIDS-defining illnesses, or (ii) are linked to an undiagnosed HIV prevalence exceeding 0.1% (routine HIV testing in such conditions is considered cost-effective when the HIV prevalence is above 0.1% [11,12]), or (iii) are conditions where an undetected HIV infection could result in serious negative outcomes for the patient.

Earlier research has confirmed the effectiveness of HIV testing guided by ICs in diverse healthcare settings [13-18]. Nevertheless, Bogers et al., in their systematic review and meta-analysis [19], emphasised the limited implementation of IC-based HIV testing in healthcare facilities across western countries (which they define as Australia, Canada, Japan, New Zealand, western Europe and the United States), noting considerable disparities in testing rates depending on the specific IC. In addition, they observed that adherence to IC-driven HIV testing has not improved over time. In European countries, fewer than half of the national medical specialty guidelines for HIV ICs have adequate HIV testing recommendations, contributing to the high levels of undiagnosed HIV and late HIV presentations observed across Europe [20].

The WHO recognises primary care (PC) as essential in achieving the goal of ending AIDS by 2030, as outlined in its Sustainable Development Goals. These goals urge countries to eliminate AIDS as a public health threat and attain universal health coverage [21]. Primary care represents a crucial setting for early HIV detection since it is the front-line service to individuals at risk or experiencing symptoms. Between 2010 and 2012, a cross-sectional, population-based study conducted in Catalonia, a region in northern Spain, found significant missed opportunities for HIV diagnosis among patients aged 16 to 65 years diagnosed with an HIV IC in a PC setting [22]. Of 99,426 patients with an HIV IC diagnosis, only 18,450 (18.56%) received an HIV test within 4 months, identifying 275 (1.49%) who tested positive. The Catalan healthcare system offers universal, free coverage to all Spanish citizens and documented migrants, with PC providing accessible services and resources for addressing common health concerns, including free HIV testing. Although general practitioners (GPs) usually manage patient care, referrals to secondary care, although not obligatory, are frequently made [23]. Spanish guidelines recommend obtaining verbal consent for HIV testing, along with providing pre-test and post-test counselling within healthcare services [24].

Medical intervention may be affected by implicit biases rooted in cultural stereotypes, perpetuating health inequities [25,26]. Implicit or unconscious bias involves associations or attitudes related to factors such as race/ethnicity, gender, socioeconomic status, age and weight, unconsciously shaping individuals' perceptions [26]. Healthcare providers are likewise vulnerable to implicit bias, which can impact their clinical judgment and patient interactions, as well as influence decision-making processes and the quality of clinician-patient interactions [27]. Gender bias, in particular, strongly shapes clinician-patient interaction [27].

This study aims to estimate the prevalence and the distribution of HIV ICs in patients attending PC in Catalonia; to evaluate the proportion of patients diagnosed with an HIV IC that were subsequently tested for HIV; to identify factors associated with not being tested for HIV for persons with an IC diagnosis, to measure the prevalence of HIV infection among tested patients with an IC diagnosis and to analyse the evolution of HIV testing based on ICs over time.

## Methods

### Study design and population

We conducted a retrospective cross-sectional population-based study using secondary data, encompassing all patients aged 16 to 65 years who were treated in PC centres operated by the Catalan Health Institute (ICS) and who had been diagnosed with an HIV IC. The ICS is the public health service in Catalonia responsible for overseeing 285 PC centres, which accounts for 95% of all PC centres in Catalonia. All data were obtained from the Information System for the Development of Research in Primary Health Care (SIDIAP), which has been validated as highly representative of the population of Catalonia in terms of geographical location, age and sex distribution [28,29]. It contains pseudoanonymised longitudinal patient information including sociodemographic information, morbidity using the International Classification of Diseases, 10th Revision (ICD-10), lifestyle and clinical variables, laboratory tests and treatments. Socioeconomic deprivation was assessed using the MEDEA socioeconomic deprivation index, developed in Spain to measure area-level deprivation based on census indicators such as unemployment, education, calculated using five census-based socioeconomic indicators (percentages, by census tract): (i) unemployment rate, (ii) proportion of manual workers, (iii) proportion of temporary workers, (iv) illiterate adults (or individuals with less than the required basic education), and (v) school drop-out rates among individuals under 16 years of age. The index classifies patients living in urban areas into five socioeconomic deprivation quintiles (Q1 (least deprived) to Q5 (most deprived)) based on place of residence, income, education and employment rate. We also included patients living in rural areas, which were defined, according to MEDEA project criteria, as municipalities with fewer than 10,000 inhabitants or a population density below 150 inhabitants per square metre. Additional details on the construction of the MEDEA index have been published elsewhere [30].

### Study period

The study period was 1 January 2017 to 31 August 2021.

### HIV indicator conditions

For the purpose of this study, an HIV IC episode was used as a study unit instead of a patient. The IC conditions studied here were based on ‘HIV Indicator Conditions: Guidance for Implementing HIV Testing in Adults in Health Care Settings’ [24], including: any AIDS-defining illness, Hepatitis B or C, sexually transmitted infections (STI), mononucleosis, lymphoma other than non-Hodgkin’s lymphoma, herpes zoster infection, seborrheic dermatitis, unexplained leukocytopenia, unexplained thrombocytopenia and candidiasis other than pulmonary or vaginal candida [31]. Identifying patients with a mononucleosis-like illness was not feasible due to the absence of specific coding in SIDIAP. However, we included patients who were diagnosed with mononucleosis or those who had a positive anti-Epstein-Barr virus IgM test or a Paul Bunnell/monospot test. All HIV ICs diagnosed between 1 January 2017 and 31 August 2021, inclusive, were considered.

### Inclusion and exclusion criteria

Patients were included in the study if aged between 16 and 65 years on the date of HIV IC diagnosis, and not pregnant or had a previous HIV diagnosis. Pregnant women were excluded to avoid including HIV tests from routine gestational screening, which is unrelated to IC-guided HIV testing and follows distinct guidelines. This exclusion prevents potential confounding, ensuring the study focuses solely on IC-guided practices.

### HIV testing definition

We defined HIV testing (yes/no) as having any HIV test within the 4 months after the date of IC diagnosis, with no new IC diagnosed between the defining IC and the HIV test. A 4-month interval was chosen to allow sufficient time for healthcare providers to schedule the test, perform the analysis and record the results. An HIV test result indicating the presence of HIV was considered a positive result. The longitudinal structure of the SIDIAP database enabled tracking the progression of patients from their initial presentation with an IC to subsequent HIV testing (or the absence thereof). By linking HIV IC diagnoses with subsequent HIV testing records, we ensured that the analysis captured the testing outcomes directly related to the IC diagnosis.

### Variables

Characteristics of patients at date of HIV IC diagnosis were collected, including sex (men, women), age group (16–30, 31–40, 41–50, 51–66 years), MEDEA socioeconomic deprivation index (five urban area quintiles and rural area), migratory status (Spanish citizen or migrant with nationality other than Spanish), sanitary health region of the assigned PC centre (Lleida, Tarragona, Barcelona, Girona, Metropolitana Sud, Metropolitana Nord, Alt Pirineu-Aran, Catalunya Central and Terres de l’Ebre) and IC diagnosis code. For multivariate analysis, ICs were classified as STIs, AIDS-defining illnesses and other.

### Statistical analysis

Characteristics of the patients at date of IC diagnosis were described annually using absolute and relative frequencies. The number of diagnosed ICs with an associated HIV test was reported using both the absolute count and the percentage within each subgroup of patient characteristics (i.e. age, sex, socioeconomic deprivation index, migratory status and region of the assigned PC centre). Frequencies were compared between these subgroups using Pearson's chi-square test. Absolute and relative frequency of positive HIV test results for each subgroup were also described; frequencies were compared between these subgroups using Pearson’s chi-square test. A multivariate logistic model was used to estimate the factors associated with HIV testing, explained by year, sex, MEDEA index, migratory status, age > 50 years, IC code and health region. Class Q3 (the middle quintile) of the MEDEA index was selected as the reference category in the multivariate analysis, as it represents the central range of socioeconomic deprivation, allowing for balanced comparisons with the most deprived (Q4, Q5) and least deprived (Q1, Q2) groups. Linear trends were explored using multiple univariate regression models with HIV testing request as dependent variable explained exclusively by the year as continuous variable, restricted to the patient subgroups. All analyses were performed using R software version 3.5.1 (R Foundation, Vienna, Austria) and a significance level of 5%.

To perform a sensitivity analysis, we replicated all the analyses, but excluded all HIV IC diagnoses that had an HIV test performed in the 4 months prior to the IC diagnosis date.

## Results

Of the 5,330,179 patients registered in SIDIAP during the study period, 292,642 patients between 16 and 65 years old experienced 372,712 episodes of a diagnosis of at least one HIV IC. Half of these diagnostic episodes involved men (48.3%), while 22.0% were in migrants, 17.4% in patients living in more socioeconomically deprived areas (Q5), 28.3% in patients residing in the Barcelona health region and 29.0% in patients over 50 years old (Supplementary Table S1). The ICs diagnosed most often were herpes zoster (17.0%), seborrheic dermatitis (14.6%) and HPV infection (12.4%). Most diagnostic episodes (97.7%) involved only one IC. Pneumonia showed the most notable change during the study period, increasing from 3.7% in 2017 to 16.4% in 2020 and 11.8% in 2021. Sexually transmitted infections also demonstrated a notable increase, with the prevalence of chlamydia, gonorrhoea and syphilis rising from 4.6%, 2.9% and 5.1%, respectively, in 2017 to 8.8%, 5.0% and 8.0%, respectively, in 2021 (Supplementary Table S2). Frequencies of each HIV IC by sex, age, migratory status, socioeconomic status and sanitary region are shown in Supplementary Tables S3–S7, respectively.

Of 372,712 diagnostic episodes of an HIV IC, 84,694 (22.7%) had an HIV test within 4 months. The proportion of patients with an IC who were tested for HIV was higher among men (26.3% compared with 19.4% for women), migrants (27.7% compared with 21.3% for Spanish nationals) and younger patients (29.2% among those aged 16–30 years compared with 13.7% for those over 50) (Table 1). Barcelona was the health region with the highest proportion of HIV testing following a diagnosis of an IC in Catalonia (28.6%). Patients residing in rural areas were less likely to be tested, with a proportion of 16.4%. For the diagnosed HIV ICs, the patients most frequently tested for HIV were those with syphilis (68.4%), genital herpes (50.6%), chlamydia (48.2%) and gonorrhoea (43.1%) (Table 2). The proportion of HIV testing in patients with an IC increased during the study period, from 20.1% in 2017 to 26.5% in 2021 (p < 0.001). Specifically, the proportion of patients tested for HIV after a diagnosis of an STI also rose during the study period: for chlamydia, gonorrhoea, syphilis and genital herpes, the rates were 45.7%, 38.3%, 64.2% and 47.1%, respectively, in 2017, increasing to 51.2%, 49.0%, 72.5% and 53.7%, respectively, in 2021 (Table 2). Other notable HIV ICs such as dermatitis and mononucleosis also saw increases from 2017 to 2021, with rates rising from 2.3% to 3.0% for dermatitis and from 32.4% to 41.1% for mononucleosis. Herpes zoster saw slight increases in 2018 (3.4%) and 2019 (3.1%) compared with 2017 (2.7%). The proportion of patients tested for HIV varied depending on the number of ICs diagnosed in the same episode, with 21.7% tested for those diagnosed with one IC, 67.1% for two ICs, 75.8% for three ICs and 53.8% for those with four to five ICs (Table 2).

**Table 1:** HIV testing in the 4 months after a diagnosis of an indicator condition, by year and demographic characteristics, Catalonia, Spain, 2017–2021

Characteristics	Global request		Global p value	2017 request		2017 p value	2018 request		2018 p value	2019 request		2019 p value	2020 request		2020 p value	2021 request		2021 p value	Trend		
Sex	n	%		n	%		n	%		n	%		n	%		n	%		OR	95% CI	p value
Women	37,354	19.4	< 0.001	6,426	17.4	< 0.001	7,733	19.7	< 0.001	8,902	20.4	< 0.001	6,649	17.7	< 0.001	7,644	21.7	< 0.001	1.04	1.03–1.05	<0.001
Men	47,340	26.3		7,663	23.1		8,921	25.1		10,730	27.1		8,989	24.7		11,037	31.2		1.08	1.08–1.09	<0.001
MEDEA																					
Rural	7,129	16.4	< 0.001	1,098	12.8	< 0.001	1,436	16.0	< 0.001	1,699	17.2	< 0.001	1,325	16.1	< 0.001	1,571	20.3	< 0.001	1.12	1.09–1.14	<0.001
Q1 (least deprived)	12,476	24.5		1,869	20.6		2,315	23.3		2,809	25.0		2,413	22.9		3,070	30.3		1.11	1.09–1.12	<0.001
Q2	12,153	23.1		1,959	20.2		2,318	22.4		2,829	24.0		2,319	21.3		2,728	27.4		1.08	1.06–1.09	<0.001
Q3	12,354	23.3		2,090	21.0		2,454	22.9		2,888	24.3		2,231	21.2		2,691	26.9		1.06	1.04–1.07	<0.001
Q4	13,762	24.0		2,274	21.4		2,709	23.4		3,148	24.7		2,528	22.2		3,103	28.2		1.07	1.05–1.08	<0.001
Q5 (most deprived)	16,050	24.7		2,714	22.5		3,122	24.4		3,785	25.9		2,930	22.6		3,499	28.0		1.05	1.04–1.06	<0.001
No information	10,770	21.4		2,085	20.6		2,300	22.0		2,474	22.2		1,892	20.0		2,019	21.9		1.00	0.99–1.02	0.610
Migrant																					
No	61,991	21.3	< 0.001	10,871	19.1	< 0.001	12,302	20.7	< 0.001	14,125	22.1	< 0.001	11,496	20.1	< 0.001	13,197	24.8	< 0.001	NA		NA
Yes	22,703	27.7		3,218	24.7		4,352	28.1		5,507	28.3		4,142	24.8		5,484	31.7				NA
Sanitary region																					
Lleida	2,845	17.1	< 0.001	548	17.0	< 0.001	556	16.6	< 0.001	660	17.2	< 0.001	498	14.6	< 0.001	583	20.5	< 0.001	1.03	1.00–1.06	0.038
Tarragona	2,981	17.6		495	15.6		632	17.6		680	17.7		595	18.0		579	19.0		1.05	1.02–1.08	<0.001
Barcelona	30,085	28.6		4,794	25.9		5,785	28.8		6,777	29.1		5,689	25.8		7,040	33.0		1.06	1.05–1.07	<0.001
Girona	5,511	17.0		1,052	16.6		1,134	16.7		1,307	17.9		921	14.8		1,097	18.8		1.02	1.00–1.04	0.110
Metropolitana Sud	18,688	25.0		3,130	22.1		3,401	22.9		4,270	25.6		3,623	24.1		4,264	30.2		1.10	1.08–1.11	<0.001
Metropolitana Nord	19,149	20.1		3,254	17.7		4,012	20.6		4,683	22.0		3,216	17.8		3,984	22.3		1.04	1.03–1.05	<0.001
Catalunya Central	4,102	19.1		524	12.7		828	18.5		971	20.5		863	20.6		916	23.3		1.16	1.13–1.19	<0.001
Alt Pirineu - Aran	230	13.9		44	13.3		55	15.3		49	12.9		39	12.8		43	15.6		1.02	0.92–1.12	0.773
Terres de l'Ebre	1,080	14.3		248	14.7		248	14.3		229	13.7		185	14.3		170	14.7		1.00	0.95–1.05	0.940
Age (years)																					
16–30	30,957	29.2	< 0.001	4,895	25.5	< 0.001	6,242	28.8	< 0.001	7,600	29.8	< 0.001	5,536	28.1	< 0.001	6,684	33.5	< 0.001	1.08	1.07–1.09	<0.001
31–40	21,386	27.1		3,759	24.2		4,327	26.7		4,912	27.7		3,853	25.8		4,535	31.3		1.07	1.06–1.08	<0.001
41–50	17,531	22.0		3,015	20.2		3,449	21.8		3,839	22.5		3,326	20.3		3,902	25.0		1.05	1.04–1.06	<0.001
51–66	14,820	13.7		2,420	11.8		2,636	12.5		3,281	14.3		2,923	12.7		3,560	17.4		1.10	1.08–1.11	<0.001

CI: confidence interval; MEDEA: *an acronym derived from Spanish: Mortalidad en áreas pequeñas Españolas y Desigualdades Socioeconómicas y Ambientales (Mortality in small Spanish areas and Socioeconomic and Environmental Inequalities)*; NA: not applicable; OR: odds ratio.

Absolute and relative frequencies (in relation to the totals observed in Supplementary Table S1) of HIV testing in the 4 months after a diagnosis of an indicator condition, and p values contrasting homogeneity in HIV testing by subgroups of the variable (globally and at each year). Last column presents the p value of a lineal trend in the evolution of such HIV testing thorough the years, accompanied by the OR (p values below 0.05 with odds ratios below 1 suggesting a decreasing trend, and p values below 0.05 with odds ratios beyond 1 suggesting an increasing trend).

**Table 2:** HIV testing in the 4 months after diagnosis of an indicator condition, by year and indicator condition, Catalonia, Spain, 2017–2020

Indicator condition	Global request		Global p value	2017 request		2017 p value	2018 request		2018 p value	2019 request		2019 p value	2020 request		2020 p value	2021 request		2021 p value	Trend		
	n	%		n	%		n	%		n	%		n	%		n	%		OR	95% CI	p value
Anal cancer	11	7.8	< 0.001	2	5.9	0.063	3	11.5	0.281	0	0.0	0.004	4	16.7	0.776	2	7.7	0.051	1.12	0.73–1.75	0.598
Cervical cancer	56	5.9	< 0.001	10	4.9	< 0.001	11	5.6	< 0.001	13	6.2	< 0.001	12	5.5	< 0.001	10	7.9	< 0.001	1.10	0.90–1.35	0.355
Candidiasis	99	13.8	< 0.001	20	12.8	0.030	23	14.7	0.031	24	15.1	0.015	16	12.2	0.017	16	13.6	0.002	0.99	0.85–1.16	0.930
Chlamydia	12,188	48.2	< 0.001	1,476	45.7	< 0.001	2,320	48.8	< 0.001	3,159	48.5	< 0.001	2,058	44.9	< 0.001	3,175	51.2	< 0.001	1.03	1.01–1.05	< 0.001
Dermatitis	1,582	2.9	< 0.001	280	2.3	< 0.001	376	3.0	< 0.001	489	3.5	< 0.001	220	2.5	< 0.001	217	3.0	< 0.001	1.04	1.00–1.08	0.039
Gonorrhoea	6,244	43.1	< 0.001	783	38.3	< 0.001	1,114	40.1	< 0.001	1,463	42.2	< 0.001	1,160	43.3	< 0.001	1,724	49.0	< 0.001	1.11	1.08–1.14	< 0.001
Granuloma inguinale	1	5.6	0.145	1	20.0	1.000	0	0.0	1.000	0	0.0	0.778	0	0.0	0.365	0	0.0	1.000	0.00	NA	0.998
Hepatitis B	4,793	19.9	< 0.001	951	19.8	0.637	1,018	19.7	< 0.001	1,150	20.3	< 0.001	714	18.6	< 0.001	960	20.9	< 0.001	1.01	0.99–1.03	0.493
Hepatitis C	6,679	34.6	< 0.001	1,402	31.1	< 0.001	1,425	34.2	< 0.001	1,528	34.3	< 0.001	1,075	38.3	< 0.001	1,249	36.9	< 0.001	1.07	1.05–1.09	< 0.001
Genital herpes	12,991	50.6	< 0.001	1,995	47.1	< 0.001	2,380	48.5	< 0.001	3,049	51.1	< 0.001	2,620	51.5	< 0.001	2,947	53.7	< 0.001	1.07	1.05–1.09	< 0.001
Herpes zoster	1,827	2.9	< 0.001	355	2.7	< 0.001	469	3.4	< 0.001	453	3.1	< 0.001	311	2.4	< 0.001	239	2.6	< 0.001	0.96	0.92–0.99	0.012
HPV infection	11,927	25.8	< 0.001	2,574	26.3	< 0.001	2,882	28.7	< 0.001	2,803	27.7	< 0.001	1,957	23.3	< 0.001	1,711	21.8	< 0.001	0.93	0.92–0.94	< 0.001
Lymphogranuloma venerum	16	35.6	0.061	1	14.3	1.000	8	80.0	< 0.001	4	30.8	0.776	3	30.0	0.764	0	0.0	0.403	0.69	0.39–1.15	0.164
Non-Hodgkin's lymphoma	178	16.4	< 0.001	34	17.7	0.459	29	13.9	0.005	37	15.7	0.006	44	21.5	0.973	34	13.9	< 0.001	0.96	0.89–1.12	0.912
Mononucleosis	10,717	36.7	< 0.001	1,649	32.4	< 0.001	1,784	33.3	< 0.001	2,161	33.5	< 0.001	2,367	42.4	< 0.001	2,756	41.1	< 0.001	1.12	1.10–1.14	< 0.001
Pneumonia	1,065	3.7	< 0.001	65	2.5	< 0.001	85	2.8	< 0.001	102	3.4	< 0.001	480	4.1	< 0.001	333	4.0	< 0.001	1.13	1.07–1.19	< 0.001
Syphilis	14,736	68.4	< 0.001	2,279	64.2	< 0.001	2,464	65.4	< 0.001	2,967	66.3	< 0.001	2,920	71.1	< 0.001	4,106	72.5	< 0.001	1.11	1.09–1.13	< 0.001
Trichomoniasis	1,884	36.6	< 0.001	323	36.0	< 0.001	416	40.8	< 0.001	522	38.2	< 0.001	279	31.4	< 0.001	344	35.2	< 0.001	0.95	0.91–0.99	0.023
Thrombocytopenia	693	6.5	< 0.001	141	4.8	< 0.001	168	6.6	< 0.001	174	7.5	< 0.001	87	6.2	< 0.001	123	8.2	< 0.001	1.12	1.06–1.19	< 0.001
Tuberculosis	288	12.9	< 0.001	63	13.6	< 0.001	55	12.0	< 0.001	79	14.7	< 0.001	52	11.4	< 0.001	39	12.7	< 0.001	0.98	0.89–1.08	0.674
Cancroid	31	40.3	< 0.001	3	23.1	1.000	7	53.8	0.016	10	71.4	< 0.001	7	30.4	0.402	4	28.6	1.000	0.92	0.66–1.29	0.629
Kaposi's sarcoma	12	25.0	0.838	3	37.5	0.432	2	28.6	1.000	3	21.4	1.000	3	37.5	0.483	1	9.1	0.334	0.75	0.45–1.22	0.253
Other defining	100	12.6	< 0.001	15	10.2	0.004	18	15.5	0.102	35	20.0	0.304	16	9.1	< 0.001	16	8.9	< 0.001	0.91	0.79–1.06	0.233
Other STIs	2,778	38.7	< 0.001	395	41.4	< 0.001	518	41.3	< 0.001	708	39.9	< 0.001	579	34.8	< 0.001	578	37.7	< 0.001	0.94	0.91–0.98	< 0.001
Number of indicator conditions																					
1	78,811	21.7	< 0.001	13,387	19.4	< 0.001	15,782	21.5	< 0.001	18,386	22.6	< 0.001	14,369	19.9	< 0.001	16,887	24.9	< 0.001	1.06	1.05–1.06	< 0.001
2	5,572	67.1		675	63.8		823	64.3		1,193	66.6		1,193	70.7		1,688	67.8		1.06	1.02–1.09	< 0.001
3	304	75.2		25	75.8		49	72.1		51	64.6		75	81.5		104	78.8		1.14	0.96–1.35	0.138
4–5	7	53.8		2	100.0		0	0.0		2	40.0		1	100.0		2	66.7		1.08	0.46–2.62	0.847

CI: confidence intervals; OR: odds ratio; STI: sexually transmitted disease.

Absolute and relative frequencies (in relation to the total cases described in Supplementary Table S2) of HIV testing in the 4 months after a diagnosis of an indicator condition, and p values contrasting the same behaviour after the diagnosis of that specific indicator condition in relation to the other indicator conditions (globally and each year). Last column presents the p value of a lineal trend in the evolution of such HIV testing thorough the years, accompanied by the OR (p values below 0.05 with odds ratios below 1 suggesting a decreasing trend, and p values below 0.05 with odds ratios beyond 1 suggesting an increasing trend).

A total of 781 (1.0%) episodes of diagnosis of at least one IC tested positive for HIV within the 4 months after the IC diagnosis (Table 3). Men were more likely to test positive than women (1.6% vs 0.2%, p < 0.001) and migrants were more likely to test positive than Spanish citizens (1.7% vs 0.7%, p < 0.001). Patients residing in the least deprived areas (Q1) had the highest proportion of positive HIV results (1.3%, p < 0.001), as did patients aged 31 to 40 years (1.3%, p < 0.001). Among the ICs diagnosed, those with the highest proportion of HIV diagnoses were: Kaposi’s sarcoma (18.2%); lymphogranuloma venereum (12.5%); syphilis (3.2%); candidiasis other than pulmonary or vaginal (3.1%); and cervical cancer (2.1%), although the absolute numbers were small (Table 4). The highest proportion of positive HIV diagnoses (3.7%, p < 0.001) were seen for three new HIV IC diagnoses in the same episode (Table 4).

**Table 3:** Positive results of HIV tests in the 4 months after a diagnosis of an indicator condition, by year and demographic characteristics, Catalonia, Spain, 2017–2021

Characteristics	Global		Global p value	2017		2017 p value	2018		2018 p value	2019		2019 p value	2020		2020 p value	2021		2021 p value
	n	%		n	%		n	%		n	%		n	%		n	%	
Overall positive results	781	1.0	0.400	115	1.0	NA	131	0.8	NA	200	1.0	NA	151	1.0	NA	184	1.0	NA
Sex																		
Women	78	0.2	< 0.001	15	0.3	< 0.001	15	0.2	< 0.001	21	0.2	< 0.001	11	0.2	< 0.001	16	0.2	< 0.001
Men	703	1.6		100	1.5		116	1.4		179	1.7		140	1.6		168	1.6	
MEDEA																		
Rural	46	0.7	< 0.001	8	1.0	0.435	9	0.7	0.056	9	0.6	< 0.001	6	0.5	0.017	14	0.9	0.042
Q1 (least deprived)	152	1.3		16	1.0		28	1.3		44	1.6		31	1.3		33	1.1	
Q2	87	0.8		15	0.9		10	0.5		14	0.5		18	0.8		30	1.1	
Q3	111	0.9		22	1.2		18	0.8		23	0.8		17	0.8		31	1.2	
Q4	111	0.8		11	0.6		16	0.6		36	1.2		25	1.0		23	0.7	
Q5 (most deprived)	129	0.9		21	1.0		26	0.9		34	0.9		25	0.9		23	0.7	
No information	145	1.4		22	1.3		24	1.1		40	1.7		29	1.6		30	1.5	
Migrant																		
No	419	0.7	< 0.001	80	0.9	0.130	64	0.6	< 0.001	107	0.8	< 0.001	75	0.7	< 0.001	93	0.7	< 0.001
Yes	362	1.7		35	1.2		67	1.6		93	1.7		76	1.9		91	1.7	
Sanitary region																		
Lleida	35	1.2	< 0.001	6	1.1	0.110	2	0.4	0.072	7	1.1	0.030	4	0.8	0.068	16	2.8	< 0.001
Tarragona	37	1.3		7	1.5		6	1.0		6	0.9		9	1.5		9	1.6	
Barcelona	358	1.2		58	1.2		65	1.1		87	1.3		67	1.2		81	1.2	
Girona	34	0.6		3	0.3		9	0.8		8	0.6		5	0.6		9	0.8	
Metropolitana Sud	197	1.1		26	0.8		23	0.7		55	1.3		41	1.1		52	1.2	
Metropolitana Nord	92	0.6		9	0.7		20	0.6		32	0.7		21	0.7		10	0.3	
Catalunya Central	18	0.5		2	0.6		3	0.5		4	0.6		3	0.4		6	0.7	
Alt Pirineu - Aran	2	1.8		0	0.0		1	3.4		0	0.0		0	0.0		1	4.5	
Terres de l'Ebre	8	0.8		4	1.7		2	0.8		1	0.4		1	0.5		0	0.0	
Age (years)																		
16–30	257	0.9	< 0.001	41	1.0	0.181	53	0.9	0.053	53	0.7	< 0.001	52	1.0	< 0.001	58	0.9	0.437
31–40	261	1.3		37	1.2		42	1.0		75	1.6		56	1.5		51	1.1	
41–50	181	1.1		25	1.0		26	0.8		56	1.5		31	1.0		43	1.1	
51–66	82	0.6		12	0.6		10	0.4		16	0.5		12	0.4		32	0.9	

MEDEA: *an acronym derived from Spanish: Mortalidad en áreas pequeñas Españolas y Desigualdades Socioeconómicas y Ambientales (Mortality in small Spanish areas and Socioeconomic and Environmental Inequalities)*; NA: not applicable; Q: quintile.

Absolute and relative frequencies (in relation to the tested cases described in Table 1) of positive results of HIV tests in the 4 months after a diagnosis of an indicator condition, and p values contrasting homogeneity in positivity by subgroups of the variable (globally and each year). No trend evaluated due to low frequencies.

**Table 4:** Positive results of HIV tests in the 4 months after a diagnosis of an indicator condition, by year and indicator condition, Catalonia, Spain, 2017–2021

Indicator conditions	Global		Global p value	2017		2017 p value	2018		2018 p value	2019		2019 p value	2020		2020 p value	2021		2021 p value
	n	%		n	%		n	%		n	%		n	%		n	%	
Other defining	2	2.0	0.576	1	7.1	0.327	0	0.0	1.000	1	2.9	0.825	0	0.0	1.000	0	0.0	1.000
Other STIs	11	0.4	0.005	3	1.0	1.000	0	0.0	0.077	0	0.0	0.010	4	0.7	0.621	4	0.7	0.607
Anal cancer	0	0.0	1.000	0	0.0	1.000	0	0.0	1.000	0	NaN	NA	0	0.0	1.000	0	0.0	1.000
Cervical cancer	1	2.1	0.963	0	0.0	1.000	0	0.0	1.000	1	9.1	0.255	0	0.0	1.000	0	0.0	1.000
Candidiasis	3	3.1	0.104	0	0.0	1.000	1	4.3	0.485	2	8.3	0.012	0	0.0	1.000	0	0.0	1.000
Chlamydia	45	0.4	< 0.001	6	0.5	0.149	7	0.3	0.007	7	0.2	< 0.001	12	0.6	0.069	13	0.4	< 0.001
Dermatitis	10	0.7	0.275	3	1.2	0.933	3	0.9	1.000	1	0.2	0.109	1	0.5	0.674	2	0.9	1.000
Gonorrhoea	62	1.0	0.725	7	1.0	1.000	14	1.3	0.117	12	0.8	0.503	13	1.1	0.685	16	0.9	0.899
Granuloma inguinale	0	0.0	1.000	0	0.0	1.000	NA	NA	NA	NA	NA	NA	NA	NA	NA	NA	NA	NA
Hepatitis B	36	0.8	0.215	2	0.2	0.032	8	0.8	1.000	5	0.5	0.066	10	1.4	0.306	11	1.2	0.720
Hepatitis C	116	1.8	< 0.001	17	1.4	0.136	18	1.3	0.051	33	2.2	< 0.001	23	2.2	< 0.001	25	2.0	< 0.001
Genital herpes	28	0.2	< 0.001	5	0.3	0.003	4	0.2	< 0.001	9	0.3	< 0.001	6	0.2	< 0.001	4	0.1	< 0.001
Herpes zoster	21	1.2	0.355	3	1.0	1.000	3	0.7	0.896	6	1.4	0.683	3	1.0	1.000	6	2.6	0.035
HPV infection	29	0.3	< 0.001	7	0.4	0.003	7	0.3	< 0.001	6	0.2	< 0.001	4	0.2	< 0.001	5	0.3	0.003
Lymphogranuloma venerum	2	12.5	< 0.001	0	0.0	1.000	1	12.5	0.094	1	25.0	0.025	0	0.0	1.000	0	NaN	NA
Non-Hodgkin's lymphoma	0	0.0	0.355	0	0.0	1.000	0	0.0	1.000	0	0.0	1.000	0	0.0	1.000	0	0.0	1.000
Mononucleosis	41	0.4	< 0.001	8	0.5	0.074	10	0.6	0.255	6	0.3	< 0.001	9	0.4	0.002	8	0.3	< 0.001
Pneumonia	3	0.3	0.039	0	0.0	0.955	0	0.0	0.845	1	1.0	1.000	1	0.2	0.147	1	0.3	0.323
Syphilis	445	3.2	< 0.001	64	3.3	< 0.001	66	2.9	< 0.001	122	4.4	< 0.001	85	3.0	< 0.001	108	2.7	< 0.001
Trichomoniasis	2	0.1	< 0.001	0	0.0	0.237	0	0.0	0.123	0	0.0	0.032	0	0.0	0.176	2	0.6	0.613
Thrombocytopenia	13	2.1	0.007	2	2.0	0.591	3	2.0	0.267	5	3.1	0.029	2	2.4	0.463	1	0.9	1.000
Tuberculosis	2	0.7	0.924	1	1.6	1.000	0	0.0	1.000	1	1.4	1.000	0	0.0	1.000	0	0.0	1.000
Cancroid	0	0.0	1.000	0	0.0	1.000	0	0.0	1.000	0	0.0	1.000	0	0.0	1.000	0	0.0	1.000
Kaposi's sarcoma	2	18.2	< 0.001	0	0.0	1.000	0	0.0	1.000	1	33.3	0.008	0	0.0	1.000	1	100.0	< 0.001
1	699	0.9	< 0.001	101	0.9	0.005	118	0.8	0.055	181	1.0	0.331	134	0.9	< 0.001	165	1.0	0.032
2	71	1.3		14	2.4		12	1.5		18	1.5		12	1.0		15	0.9	
3	11	3.7		0	0.0		1	2.1		1	2.0		5	6.8		4	3.9	
4–5	0	0.0		0	0.0		0	NaN		0	0.0		0	0.0		0	0.0	

IC: indicator condition; NaN: not a number; NA: not applicable; STI: sexually transmitted infection.

Absolute and relative frequencies (in relation to the tested cases described in Table 2) of positive results of HIV tests in the 4 months after each type of indicator condition, and p values contrasting homogeneity in positivity after the diagnose of that specific indicator condition in relation to the other indicator conditions (globally and each year). No trend evaluated due to low frequencies.

Factors associated with being tested for HIV after an episode of an IC diagnosis in PC are shown in Table 5. Men were more likely to be tested than women (odds ratio (OR) 1.59, 95% CI: 1.56 to 1.61), as were patients living in the most deprived areas (OR: 1.05, 95% CI: 1.02 to 1.08) and those in the least deprived areas (OR: 1.02, 95% CI: 0.98 to 1.05). In contrast, migrants (OR: 0.94, 95% CI: 0.92 to 0.95) and patients residing in rural areas (OR: 0.86, 95% CI: 0.88 to 0.97) were less likely to be tested for HIV compared with Spanish citizens and those living in urban areas, respectively. Similarly, patients aged over 50 years (OR: 0.68, 95% CI: 0.67 to 0.70) were less likely to be tested than younger individuals. Episodes presenting with an STI (OR: 10.16, 95% CI: 9.23 to 11.18) or an IC other than an AIDS-defining illness or an STI (OR: 2.09, 95% CI: 1.90 to 2.29) led to higher odds of being tested for HIV. There was some variability across Catalonia, with patients residing outside of Barcelona health region having lower odds of being tested for HIV after a diagnosis of an IC. Specifically, the regions of Terres de l’Ebre (OR: 0.64, 95% CI: 0.59 to 0.68), Girona (OR: 0.64, 95% CI: 0.62 to 0.66) and Tarragona (OR: 0.66, 95% CI: 0.63 to 0.69) had the lowest odds of HIV testing. Overall, the likelihood of being tested for HIV in PC in Catalonia increased over the study period compared with 2017 (Table 5).

**Table 5:** Factors associated with HIV testing in the 4 months after a diagnosis of an indicator condition; multivariate logistic regression model, Catalonia, Spain, 2017–2021

Variable	Category	OR	95% CI	p value
(Intercept)		0.06	0.05–0.06	< 0.001
Year	2017	Reference		
	2018	1.10	1.07–1.13	< 0.001
	2019	1.14	1.11–1.17	< 0.001
	2020	1.09	1.06–1.12	< 0.001
	2021	1.29	1.25–1.32	< 0.001
Sex	Women	Reference		
	Men	1.59	1.56–1.61	< 0.001
Socioeconomic environment	Rural	0.86	0.83–0.89	< 0.001
	Least deprived quintile	1.02	0.98–1.05	0.329
	Q2	0.95	0.92–0.98	0.002
	Q3	Reference		
	Q4	1.03	1.00–1.07	0.031
	Most deprived quintile	1.05	1.02–1.08	0.003
	Unclassified	0.81	0.78–0.8	< 0.001
Migrant	No	Reference		
	Yes	0.94	0.92–0.95	< 0.001
Age (years)	≤ 50	Reference		
	> 50	0.68	0.67–0.70	< 0.001
Indicator condition	No AIDS-defining illnesses	Reference		
	AIDS-defining illnesses	0.94	0.85–1.05	0.259
	No STI	Reference		
	STI	10.16	9.23–11.18	< 0.001
	No other	Reference		
	Other	2.09	1.90–2.29	< 0.001
Multiple ICs	No	Reference		
	2	2.36	2.21–2.53	< 0.001
	3	3.25	2.58–4.14	< 0.001
	4–5	1.32	0.43–4.23	0.623
Sanitary region	Lleida	0.72	0.69–0.76	< 0.001
	Tarragona	0.66	0.63–0.69	< 0.001
	Barcelona	Reference		
	Girona	0.64	0.62–0.66	< 0.001
	Metropolitana Sud	0.97	0.95–1.00	0.023
	Metropolitana Nord	0.72	0.70–0.74	< 0.001
	Catalunya Central	0.80	0.77–0.83	< 0.001
	Alt Pirineu - Aran	0.67	0.57–0.78	< 0.001
	Terres de l'Ebre	0.64	0.59–0.68	< 0.001

CI: confidence interval; IC: indicator condition; OR: odds ratio; Q: quintile; STI: sexually transmitted disease.

Odds ratios with 95% confidence intervals derived from the multivariate regression model including only the presented variables. P value contrasting the significance of the related marginal effect.

## Discussion

The results of this study highlight critical gaps and disparities in HIV testing based on IC diagnosis in PC centres in Catalonia. Despite evidence supporting the effectiveness of IC-guided HIV testing [7-9], this strategy is still poorly applied by GPs as only 22.7% of patients diagnosed with an IC were tested for HIV within 4 months. This low testing rate aligns with previous reports of missed opportunities for earlier diagnosis through IC-guided testing [22,32], and is consistent with findings from other western countries [19]. These results underscore the urgent need for enhanced efforts to improve adherence to IC-guided testing protocols in PC.

Prompt HIV diagnosis and timely linkage to care are crucial strategies for enhancing the outcomes for HIV-positive individuals and reducing the overall incidence of HIV in the general population. In many contexts, PC serves as a frontline service for patients presenting with symptoms of acute infection or those at risk of HIV. General practitioners encounter various obstacles to performing HIV tests. Key challenges identified by healthcare providers include time constraints, limited staff, assumptions of low HIV prevalence, competing health priorities (such as chronic conditions), stigma associated with HIV, insufficient sexual health education [33] and outdated HIV testing guidelines. Indicator condition-guided testing can bypass barriers faced by both patients and healthcare providers, such as obtaining a patient’s sexual history or performing HIV risk assessments [7,9]. However, previous studies have identified several reasons for the low effectiveness of HIV testing based on IC, including the lack of adequate testing protocols [34] and inadequate adherence to local testing protocols when they are available [35]. In this context, updating guidelines, combined with direct education on HIV prevalence among patients with ICs and condition-guided screening could enhance clinicians’ awareness and improve testing practices.

Several factors were identified as influencing the likelihood of HIV testing following an IC diagnosis. Notably, the type of IC substantially influenced testing rates. Patients diagnosed with an STI were 10-times more likely to be tested for HIV (OR: 10.16, 95% CI: 9.23 to 11.18), reflecting a recognition of the common co-occurrence of these conditions. However, the lower testing rates observed in patients diagnosed with other ICs, such as seborrheic dermatitis or herpes zoster, which are very common in PC, suggest that healthcare providers may not consistently recognise the association of these conditions with potential HIV infection. This indicates a need for further education and training for healthcare providers to improve recognition of the full spectrum of HIV ICs and to promote consistent testing practices.

Men, Spanish citizens, patients 30 years or younger and those living in the most urban socioeconomically deprived areas (Q5) were more likely to be tested. Patients may be impacted by implicit biases rooted in cultural stereotypes, which can reinforce existing health disparities [26,27]. The higher testing rates among younger men may reflect a perceived higher risk of HIV infection within these groups. Gender is among the traits most frequently influenced by implicit bias, notably influencing clinician-patient interactions [27]. While the higher positivity rate observed in men suggests that testing efforts are effectively targeting a key population, particularly heterosexual men, who often experience longer delays between HIV infection and diagnosis compared with gay, bisexual and other men who have sex with men (GBMSM) [1], this needs to be contextualised within the broader landscape of HIV testing. Current national and international guidelines recommend routinely offering HIV testing to women diagnosed with an IC [24,31], yet lower HIV screening rates are observed in women compared with men, both in the present study and previous studies in PC in Catalonia [22,32]. Women generally engage with healthcare services more often than men because of reproductive health requirements, childbirth, pregnancy termination services and screenings for cervical and breast cancer. However, these interactions are often missed opportunities for diagnosing HIV in women [36]. Notably, women in Catalonia experience the highest incidence of diagnostic delays, with 62.5% of cases being diagnosed late [1]. To avoid conflating gestational HIV screening with IC-guided HIV testing, pregnant women were excluded from this analysis as the two follow distinct clinical pathways. While this exclusion reduces potential confounding, it is important to note that women benefit from additional HIV testing opportunities through gestational screening. However, evidence shows they also face more missed opportunities for HIV testing outside of pregnancy and remain in the population group with the highest proportion of late HIV diagnoses in Catalonia. These findings underscore the need to improve IC-guided HIV testing strategies for women, while maintaining targeted testing efforts for men, to ensure equitable and timely diagnoses across all populations.

The lower testing rates observed in older patients and those residing in rural areas also indicate significant disparities. Older patients may not be perceived to be at high risk, despite evidence suggesting that they can have similar or even higher risk of late HIV diagnosis compared with younger individuals [1]. For older adults, implicit beliefs among practitioners regarding the epidemiological risk of HIV in middle-aged and older individuals, along with ageism, have been recognised as reasons why general practitioners may neglect the HIV risk in this age group [37]. The lower testing rates in rural areas may be attributed to lower awareness of HIV testing guidelines among healthcare providers in these regions. Healthcare providers may also hold negative implicit biases towards individuals from lower social classes [25], including assumptions that these individuals are at higher risk of HIV exposure. This may account for the increased likelihood of HIV testing among patients residing in the most deprived areas (Q5) in our study while no significant difference was found between the least deprived areas (Q1) and the reference area (Q3).

Although migrants had a lower probability of HIV testing compared with Spanish citizens (OR: 0.94, p < 0.0001), this difference was no longer significant after adjusting for the type of diagnosed ICs in the multivariate model. Migrants accounted for 60% of the STI diagnoses included in the study, which were the ICs for which HIV testing was most frequently performed. In contrast, migrants contributed only 32% of ICs other than STI and AIDS-defining illnesses, such as seborrheic dermatitis, herpes zoster, mononucleosis-like illness, for which HIV testing was less frequently performed. Spanish citizens, by comparison, contributed 51% of these non-STI, non-AIDS-defining ICs. Overall, the probability of HIV testing was lower following a diagnosis of these ICs.

However, it is important to acknowledge that the dataset used in this study does not include direct measures or indicators of implicit bias among healthcare providers, nor does it capture the decision-making processes underlying the observed testing patterns. As such, while the hypothesis of implicit bias is supported by existing literature and aligns with the disparities observed in our study, it remains a speculative explanation rather than a definitive conclusion. This hypothesis provides a framework to contextualise our findings, emphasising the need for future research to directly assess the role of implicit biases through qualitative studies, experimental designs or datasets that incorporate provider-level and decision-making variables.

The study also revealed geographic disparities in HIV testing rates across Catalonia, with the Barcelona health region exhibiting the highest testing rate. This suggests that local healthcare policies, resource availability and possibly regional awareness campaigns might play an important role in influencing testing rates (28.6% in Barcelona compared with 13.9% in Alt Pirineu-Aran, a rural area in the Pyrenees Mountains, for example). Regions with lower testing rates may benefit from targeted interventions to improve HIV testing awareness and practices.

The increase in HIV testing rates throughout the study period represents a favourable trend, indicating growing adherence to IC-guided testing protocols. HIV testing rates rose from 20.1% in 2017 to 26.5% in 2021 (p < 0.001), although there was a decrease in 2020 probably as a consequence of the COVID-19 pandemic. The extensive reorganisation of healthcare systems during the pandemic negatively impacted all screening campaigns and non-severe acute respiratory syndrome coronavirus 2-related activities [38]. Nevertheless, despite the rise in HIV testing based on IC, the overall low testing rate and observed disparities indicate meaningful room for improvement. To enhance HIV testing rates, strategies should include targeted education and training for healthcare providers, especially in rural areas and among older populations and women, as well as public health campaigns to increase awareness of the importance of HIV screening.

Our findings show that the HIV prevalence in patients presenting to PC in Catalonia with at least one of the majority of ICs included in the present study exceeds the 0.1% threshold considered to be cost effective [11,12]. This overall observation does not include certain ICs that are rarely encountered in PC. The highest HIV prevalence was found among patients diagnosed with Kaposi’s sarcoma (18.2%), lymphogranuloma venereum (12.5%), syphilis (3.2%) and candidiasis other than pulmonary or vaginal (3.1%). Regarding the 781 HIV-positive patients identified in our analysis (0.40%), their demographic characteristics match those of the newly diagnosed patients according to Spanish and European data, with young men under the age of 40 years being the most affected group and 46.3% originating from countries other than Spain [1,2].

The study's strengths include its large, representative sample size and the use of comprehensive, routinely collected healthcare data. However, there are some limitations. Only primary care teams within the ICS in Catalonia were included, so the findings may not be applicable to all PC settings across Catalonia, even though ICS covers 95% of the total PC centres in the region. The number of missed HIV testing opportunities in PC may be overestimated because of insufficient data on test refusal rates or IC diagnoses documented by GPs but actually determined by other specialists. However, the study demonstrated that for ICs usually handled in PC settings (such as herpes zoster, seborrheic dermatitis and infectious mononucleosis), the proportion of patients who underwent HIV testing within 4 months was quite low. Furthermore, our data collection did not include HIV tests conducted outside the ICS healthcare system. The lack of a specific ICD code for mononucleosis-like illness meant that we could not identify patients with this particularly relevant IC. As previously mentioned, while disparities in HIV testing following IC diagnoses are evident, the role of implicit bias is inferred from existing literature rather than being directly supported by our data. In addition, limited socioeconomic variables and the absence of provider-specific information on training, attitudes, or decision-making processes constrain our ability to identify underlying causes.

## Conclusion

Our study reveals important gaps in HIV testing based on IC in PC in Catalonia, leading to numerous missed opportunities for early diagnosis and treatment. Enhancing education and training for healthcare providers, as well as addressing demographic and geographic disparities, are key steps to improving adherence to testing guidelines. It is essential to train professionals to recognise HIV ICs, understand when they should prompt an HIV test and apply this knowledge in clinical practice by following established guidelines. This also involves providing tools to overcome implicit bias and prevent delayed HIV diagnoses. These strategies can notably increase early HIV diagnosis and treatment, which are vital to achieving the United Nation’s 95–95–95 goals of 95% of people living with HIV know their status, 95% of those diagnosed receive treatment and 95% of those treated achieve viral suppression, by 2030, ultimately reducing HIV incidence.

## Data Availability

The raw data supporting the conclusions of this article will be made available by the authors upon reasonable request. Interested researchers can contact the corresponding author via email to access the data. Data sharing will comply with applicable privacy regulations and ethical considerations.

## Supplementary Data

498000 Supplementary Material
