# Supplementary material for: Implicit bias in HIV testing based on indicator conditions in primary care: a population-based study in Catalonia, Spain, 2017 to 2021
Source: Euro Surveill. 2025 Jun 19;30(24):2400585. doi: 10.2807/1560-7917.ES.2025.30.24.2400585 (PMC12180294; doi:10.2807/1560-7917.ES.2025.30.24.2400585)
Supplement: Supplementary Material [file 24-00585_AGUSTI_Supplement.pdf]

This supplementary material is hosted by *Eurosurveillance* as supporting information alongside the article ‘Implicit bias in HIV testing based on indicator conditions in primary care: a population-based study in Catalonia, Spain, 2017 to 2021’, on behalf of the authors, who remain responsible for the accuracy and appropriateness of the content. The same standards for ethics, copyright, attributions and permissions as for the article apply. Supplements are not edited by *Eurosurveillance* and the journal is not responsible for the maintenance of any links or email addresses provided therein.

Supplementary Table S1. Sociodemographic characteristics around the diagnoses of an indicator condition, by year

	Global	2017 (n=70,070)	2018 (n=74,810)	2019 (n=83,266)	2020 (n=74,033)	2021 (n=70,533)	p-value
Sex							<0.001
Women	192,823 (51.7%)	36,966 (52.8%)	39,338 (52.6%)	43,714 (52.5%)	37,637 (50.8%)	35,168 (49.9%)	
Men	179,889 (48.3%)	33,104 (47.2%)	35,472 (47.4%)	39,552 (47.5%)	36,396 (49.2%)	35,365 (50.1%)	
MEDEA							<0.001
Rural	43,380 (11.6%)	8,582 (12.2%)	8,970 (12.0%)	9,850 (11.8%)	8,233 (11.1%)	7,745 (11.0%)	
Q1 (less deprivation)	50,869 (13.6%)	9,068 (12.9%)	9,933 (13.3%)	11,230 (13.5%)	10,515 (14.2%)	10,123 (14.4%)	
Q2	52,680 (14.1%)	9,680 (13.8%)	10,354 (13.8%)	11,785 (14.2%)	10,900 (14.7%)	9,961 (14.1%)	
Q3	53,069 (14.2%)	9,931 (14.2%)	10,713 (14.3%)	11,882 (14.3%)	10,548 (14.2%)	9,995 (14.2%)	
Q4	57,351 (15.4%)	10,620 (15.2%)	11,560 (15.5%)	12,761 (15.3%)	11,407 (15.4%)	11,003 (15.6%)	
Q5 (greater deprivation)	64,929 (17.4%)	12,052 (17.2%)	12,806 (17.1%)	14,626 (17.6%)	12,954 (17.5%)	12,491 (17.7%)	
No information	50,434 (13.5%)	10,137 (14.5%)	10,474 (14.0%)	11,132 (13.4%)	9,476 (12.8%)	9,215 (13.1%)	
Migrant							<0.001
No	290,814 (78.0%)	57,065 (81.4%)	59,337 (79.3%)	63,827 (76.7%)	57,327 (77.4%)	53,258 (75.5%)	
Yes	81,898 (22.0%)	13,005 (18.6%)	15,473 (20.7%)	19,439 (23.3%)	16,706 (22.6%)	17,275 (24.5%)	
Sanitary Region							<0.001
Lleida	16,672 (4.5%)	3,233 (4.6%)	3,351 (4.5%)	3,846 (4.6%)	3,404 (4.6%)	2,838 (4.0%)	
Tarragona	16,970 (4.6%)	3,181 (4.5%)	3,595 (4.8%)	3,844 (4.6%)	3,305 (4.5%)	3,045 (4.3%)	
Barcelona	105,274 (28.3%)	18,533 (26.5%)	20,058 (26.9%)	23,325 (28.1%)	22,045 (29.8%)	21,313 (30.3%)	
Girona	32,493 (8.7%)	6,356 (9.1%)	6,773 (9.1%)	7,315 (8.8%)	6,216 (8.4%)	5,833 (8.3%)	
Metropolitana Sud	74,862 (20.1%)	14,153 (20.2%)	14,876 (19.9%)	16,669 (20.1%)	15,061 (20.4%)	14,103 (20.0%)	
Metropolitana Nord	95,067 (25.6%)	18,356 (26.2%)	19,437 (26.0%)	21,299 (25.6%)	18,087 (24.5%)	17,888 (25.4%)	
Catalunya Central	21,499 (5.8%)	4,138 (5.9%)	4,481 (6.0%)	4,748 (5.7%)	4,193 (5.7%)	3,939 (5.6%)	
Alt Pirineu - Aran	1,651 (0.4%)	332 (0.5%)	360 (0.5%)	379 (0.5%)	305 (0.4%)	275 (0.4%)	
Terres de l'Ebre	7,545 (2.0%)	1,690 (2.4%)	1,734 (2.3%)	1,673 (2.0%)	1,290 (1.7%)	1,158 (1.6%)	
Age							<0.001
16 - 30	106,082 (28.5%)	19,206 (27.4%)	21,672 (29.0%)	25,511 (30.6%)	19,729 (26.6%)	19,964 (28.3%)	
31 - 40	78,876 (21.2%)	15,504 (22.1%)	16,235 (21.7%)	17,728 (21.3%)	14,916 (20.1%)	14,493 (20.5%)	
41 - 50	79,839 (21.4%)	14,906 (21.3%)	15,851 (21.2%)	17,089 (20.5%)	16,401 (22.2%)	15,592 (22.1%)	
51- 66	107,915 (29.0%)	20,454 (29.2%)	21,052 (28.1%)	22,938 (27.5%)	22,987 (31.0%)	20,484 (29.0%)	

Absolute and relative frequencies of the different characteristics in the total diagnostic episodes including an indicator condition, globally, and at each year. P-values contrast homogeneity along years in the distribution of the variables.

Supplementary Table S2. Frequency of each type of Indicator conditions in the sample of diagnostic episodes, by year

	Global	2017 (n=70,070)	2018 (n=74,810)	2019 (n=83,266)	2020 (n=74,033)	2021 (n=70,533)	p-value
Anal Cancer	141 (0.04%)	34 (0.05%)	26 (0.03%)	31 (0.04%)	24 (0.03%)	26 (0.04%)	0.574
Cervical cancer	956 (0.26%)	205 (0.29%)	197 (0.26%)	210 (0.25%)	218 (0.29%)	126 (0.18%)	<0.001
Candidiasis	720 (0.19%)	156 (0.22%)	156 (0.21%)	159 (0.19%)	131 (0.18%)	118 (0.17%)	0.109
Chlamydia	25,292 (6.79%)	3,232 (4.61%)	4,755 (6.36%)	6,515 (7.82%)	4,588 (6.20%)	6,202 (8.79%)	<0.001
Dermatitis	54,579 (14.64%)	12,057 (17.21%)	12,605 (16.85%)	13,860 (16.65%)	8,817 (11.91%)	7,240 (10.26%)	<0.001
Gonorrhea	14,491 (3.89%)	2,046 (2.92%)	2,779 (3.71%)	3,468 (4.16%)	2,678 (3.62%)	3,520 (4.99%)	<0.001
Granuloma inguinale	18 (0.00%)	5 (0.01%)	2 (0.00%)	3 (0.00%)	7 (0.01%)	1 (0.00%)	0.151
Hepatitis B	24,037 (6.45%)	4,795 (6.84%)	5,157 (6.89%)	5,658 (6.80%)	3,841 (5.19%)	4,586 (6.50%)	<0.001
Hepatitis C	19,327 (5.19%)	4,503 (6.43%)	4,172 (5.58%)	4,461 (5.36%)	2,808 (3.79%)	3,383 (4.80%)	<0.001
Genital herpes	25,686 (6.89%)	4,233 (6.04%)	4,910 (6.56%)	5,970 (7.17%)	5,086 (6.87%)	5,487 (7.78%)	<0.001
Herpes zoster	63,547 (17.05%)	13,267 (18.93%)	13,623 (18.21%)	14,403 (17.30%)	12,948 (17.49%)	9,306 (13.19%)	<0.001
HPV infection	46,193 (12.39%)	9,779 (13.96%)	10,046 (13.43%)	10,135 (12.17%)	8,402 (11.35%)	7,831 (11.10%)	<0.001
Lymphogranuloma venereal	45 (0.01%)	7 (0.01%)	10 (0.01%)	13 (0.02%)	10 (0.01%)	5 (0.01%)	0.592
No Hodgkin's lymphoma	1,085 (0.29%)	192 (0.27%)	208 (0.28%)	235 (0.28%)	205 (0.28%)	245 (0.35%)	0.048
Mononucleosis	29,171 (7.83%)	5,091 (7.27%)	5,352 (7.15%)	6,442 (7.74%)	5,582 (7.54%)	6,704 (9.50%)	<0.001
Pneumonia	28,851 (7.74%)	2,625 (3.75%)	3,043 (4.07%)	2,991 (3.59%)	11,850 (16.01%)	8,342 (11.83%)	<0.001
Syphilis	21,555 (5.78%)	3,550 (5.07%)	3,766 (5.03%)	4,474 (5.37%)	4,105 (5.54%)	5,660 (8.02%)	<0.001
Trichomones	5,150 (1.38%)	897 (1.28%)	1,020 (1.36%)	1,367 (1.64%)	888 (1.20%)	978 (1.39%)	<0.001
Thrombocytopenia	10,697 (2.87%)	2,939 (4.19%)	2,556 (3.42%)	2,318 (2.78%)	1,392 (1.88%)	1,492 (2.12%)	<0.001
Tuberculosis	2,226 (0.60%)	464 (0.66%)	460 (0.61%)	539 (0.65%)	457 (0.62%)	306 (0.43%)	<0.001
Xancroid	77 (0.02%)	13 (0.02%)	13 (0.02%)	14 (0.02%)	23 (0.03%)	14 (0.02%)	0.283
Kaposi's sarcoma	48 (0.01%)	8 (0.01%)	7 (0.01%)	14 (0.02%)	8 (0.01%)	11 (0.02%)	0.647
Other defining	793 (0.21%)	147 (0.21%)	116 (0.16%)	175 (0.21%)	175 (0.24%)	180 (0.26%)	<0.001
Other STIs	7,182 (1.93%)	955 (1.36%)	1,253 (1.67%)	1,774 (2.13%)	1,665 (2.25%)	1,535 (2.18%)	<0.001
Number of IC							<0.001
1	363,988 (97.66%)	68,977 (98.44%)	73,460 (98.20%)	81,392 (97.75%)	72,252 (97.59%)	67,907 (96.28%)	
2	8,307 (2.23%)	1,058 (1.51%)	1,280 (1.71%)	1,790 (2.15%)	1,688 (2.28%)	2,491 (3.53%)	
3	404 (0.11%)	33 (0.05%)	68 (0.09%)	79 (0.09%)	92 (0.12%)	132 (0.19%)	
4-5	13 (0.00%)	2 (0.00%)	2 (0.00%)	5 (0.01%)	1 (0.00%)	3 (0.00%)	

Absolute and relative frequencies of the different indicator conditions in the total diagnostic episodes including an indicator condition, globally, and at each year. P-values contrast homogeneity along years in the distribution of the relative presence of such indicator condition.

Supplementary Table S3. Frequency of each type of Indicator conditions in the sample of diagnostic episodes, by sex.

	Women (n=192,823)	Men (n=179,889)	p-value
Anal Cancer	71 (0.04%)	70 (0.04%)	0.807
Cervical cancer	956 (0.50%)	0 (0.00%)	<0.001
Candidiasis	399 (0.21%)	321 (0.18%)	0.052
Chlamydia	16,341 (8.47%)	8,951 (4.98%)	<0.001
Dermatitis	26,927 (13.96%)	27,652 (15.37%)	<0.001
Gonorrhea	3,759 (1.95%)	10,732 (5.97%)	<0.001
Granuloma inguinale	11 (0.01%)	7 (0.00%)	0.575
Hepatitis B	8,940 (4.64%)	15,097 (8.39%)	<0.001
Hepatitis C	6,319 (3.28%)	13,008 (7.23%)	<0.001
Genital herpes	15,241 (7.90%)	10,445 (5.81%)	<0.001
Herpes zoster	37,495 (19.45%)	26,052 (14.48%)	<0.001
HPV infection	32,407 (16.81%)	13,786 (7.66%)	<0.001
Lymphogranuloma venereal	2 (0.00%)	43 (0.02%)	<0.001
No Hodgkin's lymphoma	470 (0.24%)	615 (0.34%)	<0.001
Mononucleosis	15,912 (8.25%)	13,259 (7.37%)	<0.001
Pneumonia	13,006 (6.75%)	15,845 (8.81%)	<0.001
Syphilis	3,762 (1.95%)	17,793 (9.89%)	<0.001
Trichomones	4,772 (2.47%)	378 (0.21%)	<0.001
Thrombocytopenia	4,898 (2.54%)	5,799 (3.22%)	<0.001
Tuberculosis	823 (0.43%)	1,403 (0.78%)	<0.001
Xancroid	12 (0.01%)	65 (0.04%)	<0.001
Kaposi's sarcoma	7 (0.00%)	41 (0.02%)	<0.001
Other defining	342 (0.18%)	451 (0.25%)	<0.001
Other STIs	3,451 (1.79%)	3,731 (2.07%)	<0.001

Absolute and relative frequencies of the different indicator conditions in the total diagnostic episodes including an indicator condition, globally, and by sex. P-values contrast homogeneity in the distribution of the relative presence of such indicator condition between sex groups.

Supplementary Table S4. Frequency of each type of Indicator conditions in the sample of diagnostic episodes, by age.

	[16,30] (n=106,082)	(30,40] (n=78,876)	(40,50] (n=79,839)	(50,66] (n=107,915)	p-value
Anal Cancer	3 (0.00%)	6 (0.01%)	23 (0.03%)	109 (0.10%)	<0.001
Cervical cancer	53 (0.05%)	211 (0.27%)	304 (0.38%)	388 (0.36%)	<0.001
Candidiasis	82 (0.08%)	102 (0.13%)	168 (0.21%)	368 (0.34%)	<0.001
Chlamydia	17,774 (16.75%)	4,784 (6.07%)	2,127 (2.66%)	607 (0.56%)	<0.001
Dermatitis	16,777 (15.82%)	11,180 (14.17%)	10,519 (13.18%)	16,103 (14.92%)	<0.001
Gonorrhea	7,549 (7.12%)	3,995 (5.06%)	2,031 (2.54%)	916 (0.85%)	<0.001
Granuloma inguinale	8 (0.01%)	3 (0.00%)	4 (0.01%)	3 (0.00%)	0.435
Hepatitis B	3,278 (3.09%)	5,974 (7.57%)	6,693 (8.38%)	8,092 (7.50%)	<0.001
Hepatitis C	998 (0.94%)	3,049 (3.87%)	5,767 (7.22%)	9,513 (8.82%)	<0.001
Genital herpes	9,167 (8.64%)	6,158 (7.81%)	5,053 (6.33%)	5,308 (4.92%)	<0.001
Herpes zoster	8,824 (8.32%)	9,510 (12.06%)	13,445 (16.84%)	31,768 (29.44%)	<0.001
HPV infection	15,250 (14.38%)	12,880 (16.33%)	11,516 (14.42%)	6,547 (6.07%)	<0.001
Lymphogranuloma venereal	19 (0.02%)	17 (0.02%)	7 (0.01%)	2 (0.00%)	<0.001
No Hodgkin's lymphoma	92 (0.09%)	140 (0.18%)	236 (0.30%)	617 (0.57%)	<0.001
Mononucleosis	12,710 (11.98%)	5,399 (6.84%)	4,909 (6.15%)	6,153 (5.70%)	<0.001
Pneumonia	2,514 (2.37%)	4,769 (6.05%)	7,777 (9.74%)	13,791 (12.78%)	<0.001
Syphilis	5,161 (4.87%)	7,016 (8.89%)	5,631 (7.05%)	3,747 (3.47%)	<0.001
Trichomones	1,591 (1.50%)	1,504 (1.91%)	1,460 (1.83%)	595 (0.55%)	<0.001
Thrombocytopenia	1,843 (1.74%)	1,874 (2.38%)	2,341 (2.93%)	4,639 (4.30%)	<0.001
Tuberculosis	615 (0.58%)	495 (0.63%)	560 (0.70%)	556 (0.52%)	<0.001
Xancroid	36 (0.03%)	23 (0.03%)	11 (0.01%)	7 (0.01%)	<0.001
Kaposi's sarcoma	3 (0.00%)	6 (0.01%)	7 (0.01%)	32 (0.03%)	<0.001
Other defining	198 (0.19%)	152 (0.19%)	187 (0.23%)	256 (0.24%)	0.022
Other STIs	4,194 (3.95%)	1,634 (2.07%)	959 (1.20%)	395 (0.37%)	<0.001

Absolute and relative frequencies of the different indicator conditions in the total diagnostic episodes including an indicator condition, globally, and by age. P-values contrast homogeneity in the distribution of the relative presence of such indicator condition between age groups.

Supplementary Table S5. Frequency of each type of Indicator conditions in the sample of diagnostic episodes, by migrant status.

	Spain (n=290,814)	Migrant (n=81,898)	p-value
Anal Cancer	128 (0.04%)	13 (0.02%)	<0.001
Cervical cancer	691 (0.24%)	265 (0.32%)	<0.001
Candidiasis	621 (0.21%)	99 (0.12%)	<0.001
Chlamydia	18,682 (6.42%)	6,610 (8.07%)	<0.001
Dermatitis	44,215 (15.20%)	10,364 (12.65%)	<0.001
Gonorrhea	9,837 (3.38%)	4,654 (5.68%)	<0.001
Granuloma inguinale	15 (0.01%)	3 (0.00%)	0.796
Hepatitis B	11,460 (3.94%)	12,577 (15.36%)	<0.001
Hepatitis C	14,553 (5.00%)	4,774 (5.83%)	<0.001
Genital herpes	20,024 (6.89%)	5,662 (6.91%)	0.786
Herpes zoster	56,640 (19.48%)	6,907 (8.43%)	<0.001
HPV infection	36,806 (12.66%)	9,387 (11.46%)	<0.001
Lymphogranuloma venereal	29 (0.01%)	16 (0.02%)	0.043
No Hodgkin's lymphoma	960 (0.33%)	125 (0.15%)	<0.001
Mononucleosis	26,197 (9.01%)	2,974 (3.63%)	<0.001
Pneumonia	23,915 (8.22%)	4,936 (6.03%)	<0.001
Syphilis	13,405 (4.61%)	8,150 (9.95%)	<0.001
Trichomones	3,391 (1.17%)	1,759 (2.15%)	<0.001
Thrombocytopenia	9,051 (3.11%)	1,646 (2.01%)	<0.001
Tuberculosis	1,025 (0.35%)	1,201 (1.47%)	<0.001
Xancroid	56 (0.02%)	21 (0.03%)	0.324
Kaposi's sarcoma	35 (0.01%)	13 (0.02%)	0.496
Other defining	507 (0.17%)	286 (0.35%)	<0.001
Other STIs	5,349 (1.84%)	1,833 (2.24%)	<0.001

Absolute and relative frequencies of the different indicator conditions in the total diagnostic episodes including an indicator condition, globally, and by migrant status. P-values contrast homogeneity in the distribution of the relative presence of such indicator condition between migrants and non-migrants.

Supplementary Table S6. Frequency of each type of Indicator conditions in the sample of diagnostic episodes, by socioeconomic status.

	R (n=43,380)	U1 (n=50,869)	U2 (n=52,680)	U3 (n=53,069)	U4 (n=57,351)	U5 (n=64,929)	Unclassified (n=50,434)	p-value
Anal Cancer	14 (0.03%)	25 (0.05%)	25 (0.05%)	11 (0.02%)	18 (0.03%)	17 (0.03%)	31 (0.06%)	0.007
Cervical cancer	129 (0.30%)	113 (0.22%)	124 (0.24%)	142 (0.27%)	139 (0.24%)	142 (0.22%)	167 (0.33%)	0.002
Candidiasis	113 (0.26%)	88 (0.17%)	84 (0.16%)	103 (0.19%)	100 (0.17%)	118 (0.18%)	114 (0.23%)	0.005
Chlamydia	2,113 (4.87%)	3,719 (7.31%)	3,899 (7.40%)	3,543 (6.68%)	3,923 (6.84%)	4,453 (6.86%)	3,642 (7.22%)	<0.001
Dermatitis	7,350 (16.94%)	6,467 (12.71%)	7,593 (14.41%)	8,217 (15.48%)	8,894 (15.51%)	9,936 (15.30%)	6,122 (12.14%)	<0.001
Gonorrhea	972 (2.24%)	2,518 (4.95%)	2,150 (4.08%)	1,913 (3.60%)	2,184 (3.81%)	2,479 (3.82%)	2,275 (4.51%)	<0.001
Granuloma inguinale	3 (0.01%)	2 (0.00%)	2 (0.00%)	0 (0.00%)	2 (0.00%)	4 (0.01%)	5 (0.01%)	0.390
Hepatitis B	2,245 (5.18%)	2,140 (4.21%)	2,860 (5.43%)	3,224 (6.08%)	3,972 (6.93%)	6,297 (9.70%)	3,299 (6.54%)	<0.001
Hepatitis C	1,733 (3.99%)	2,066 (4.06%)	2,221 (4.22%)	2,455 (4.63%)	2,991 (5.22%)	4,367 (6.73%)	3,494 (6.93%)	<0.001
Genital herpes	2,120 (4.89%)	3,698 (7.27%)	3,846 (7.30%)	3,727 (7.02%)	4,084 (7.12%)	4,355 (6.71%)	3,856 (7.65%)	<0.001
Herpes zoster	10,512 (24.23%)	9,124 (17.94%)	9,304 (17.66%)	9,460 (17.83%)	9,167 (15.98%)	8,808 (13.57%)	7,172 (14.22%)	<0.001
HPV infection	5,095 (11.75%)	5,969 (11.73%)	6,753 (12.82%)	6,760 (12.74%)	7,448 (12.99%)	7,984 (12.30%)	6,184 (12.26%)	<0.001
Lymphogranuloma venereal	2 (0.00%)	9 (0.02%)	9 (0.02%)	1 (0.00%)	6 (0.01%)	14 (0.02%)	4 (0.01%)	0.023
No Hodgkin's lymphoma	187 (0.43%)	142 (0.28%)	144 (0.27%)	136 (0.26%)	126 (0.22%)	147 (0.23%)	203 (0.40%)	<0.001
Mononucleosis	3,252 (7.50%)	4,505 (8.86%)	4,150 (7.88%)	4,296 (8.10%)	4,430 (7.72%)	3,794 (5.84%)	4,744 (9.41%)	<0.001
Pneumonia	3,585 (8.26%)	4,287 (8.43%)	4,255 (8.08%)	4,119 (7.76%)	4,299 (7.50%)	5,002 (7.70%)	3,304 (6.55%)	<0.001
Syphilis	1,222 (2.82%)	3,964 (7.79%)	2,825 (5.36%)	2,777 (5.23%)	2,957 (5.16%)	3,683 (5.67%)	4,127 (8.18%)	<0.001
Trichomones	479 (1.10%)	498 (0.98%)	652 (1.24%)	701 (1.32%)	958 (1.67%)	1,181 (1.82%)	681 (1.35%)	<0.001
Thrombocytopenia	1,742 (4.02%)	1,418 (2.79%)	1,481 (2.81%)	1,426 (2.69%)	1,589 (2.77%)	1,733 (2.67%)	1,308 (2.59%)	<0.001
Tuberculosis	253 (0.58%)	217 (0.43%)	263 (0.50%)	299 (0.56%)	350 (0.61%)	574 (0.88%)	270 (0.54%)	<0.001
Xancroid	12 (0.03%)	6 (0.01%)	13 (0.02%)	16 (0.03%)	16 (0.03%)	8 (0.01%)	6 (0.01%)	0.082
Kaposi's sarcoma	5 (0.01%)	10 (0.02%)	6 (0.01%)	7 (0.01%)	3 (0.01%)	8 (0.01%)	9 (0.02%)	0.474
Other defining	70 (0.16%)	128 (0.25%)	125 (0.24%)	86 (0.16%)	117 (0.20%)	141 (0.22%)	126 (0.25%)	0.002
Other STIs	842 (1.94%)	982 (1.93%)	1,148 (2.18%)	956 (1.80%)	1,047 (1.83%)	1,269 (1.95%)	938 (1.86%)	<0.001

Absolute and relative frequencies of the different indicator conditions in the total diagnostic episodes including an indicator condition, globally, and by MEDEA deprivation quintiles (R: Rural, U1: least deprived socioeconomic quintile, U5 most deprived socioeconomic quintile). P-values contrast homogeneity in the distribution of the relative presence of such indicator condition between socioeconomic subgroups.

Supplementary Table S7. Frequency of each type of Indicator condition in the sample of diagnostic episodes, by sanitary region.

	Lleida (n=16,672)	Tarragona (n=16,970)	Barcelona (n=105,274)	Girona (n=32,493)	Metropolitana Sud (n=74,862)	Metropolitana Nord (n=95,067)	Catalunya Central (n=21,499)	Alt Pirineu – Aran (n=1,651)	Terres de l'Ebre (n=7,545)	p-value
Candidiasis	33 (0.20%)	39 (0.23%)	179 (0.17%)	85 (0.26%)	169 (0.23%)	145 (0.15%)	51 (0.24%)	5 (0.30%)	14 (0.19%)	<0.001
Chlamydia	836 (5.01%)	692 (4.08%)	9,311 (8.84%)	1,228 (3.78%)	4,897 (6.54%)	6,684 (7.03%)	1,328 (6.18%)	51 (3.09%)	201 (2.66%)	<0.001
Dermatitis	2,805 (16.82%)	3,352 (19.75%)	12,776 (12.14%)	5,842 (17.98%)	11,699 (15.63%)	12,810 (13.47%)	3,601 (16.75%)	245 (14.84%)	1,446 (19.17%)	<0.001
Gonorrhea	578 (3.47%)	739 (4.35%)	6,853 (6.51%)	762 (2.35%)	2,587 (3.46%)	2,307 (2.43%)	478 (2.22%)	28 (1.70%)	131 (1.74%)	<0.001
Granuloma inguinale	1 (0.01%)	4 (0.02%)	4 (0.00%)	3 (0.01%)	2 (0.00%)	1 (0.00%)	0 (0.00%)	2 (0.12%)	1 (0.01%)	<0.001
Hepatitis B	945 (5.67%)	421 (2.48%)	6,351 (6.03%)	3,479 (10.71%)	4,972 (6.64%)	5,981 (6.29%)	1,454 (6.76%)	98 (5.94%)	227 (3.01%)	<0.001
Hepatitis C	969 (5.81%)	360 (2.12%)	6,465 (6.14%)	1,766 (5.44%)	4,151 (5.54%)	4,208 (4.43%)	1,009 (4.69%)	65 (3.94%)	241 (3.19%)	<0.001
Genital herpes	656 (3.93%)	755 (4.45%)	8,819 (8.38%)	1,349 (4.15%)	6,281 (8.39%)	6,164 (6.48%)	1,167 (5.43%)	70 (4.24%)	341 (4.52%)	<0.001
Herpes zoster	3,597 (21.58%)	3,631 (21.40%)	13,462 (12.79%)	6,873 (21.15%)	13,374 (17.86%)	15,510 (16.31%)	4,637 (21.57%)	455 (27.56%)	2,001 (26.52%)	<0.001
HPV infection	1,602 (9.61%)	3,055 (18.00%)	12,230 (11.62%)	3,192 (9.82%)	7,685 (10.27%)	14,891 (15.66%)	2,265 (10.54%)	148 (8.96%)	1,035 (13.72%)	<0.001
Lymphogranuloma venereal	1 (0.01%)	2 (0.01%)	33 (0.03%)	2 (0.01%)	3 (0.00%)	3 (0.00%)	1 (0.00%)	0 (0.00%)	0 (0.00%)	<0.001
No Hodgkin's lymphoma	77 (0.46%)	79 (0.47%)	238 (0.23%)	104 (0.32%)	225 (0.30%)	244 (0.26%)	69 (0.32%)	10 (0.61%)	39 (0.52%)	<0.001
Mononucleosis	1,239 (7.43%)	925 (5.45%)	7,220 (6.86%)	1,388 (4.27%)	7,779 (10.39%)	8,381 (8.82%)	1,277 (5.94%)	144 (8.72%)	616 (8.16%)	<0.001
Pneumonia	1,568 (9.40%)	1,182 (6.97%)	8,266 (7.85%)	2,319 (7.14%)	5,357 (7.16%)	7,423 (7.81%)	1,961 (9.12%)	183 (11.08%)	587 (7.78%)	<0.001
Syphilis	552 (3.31%)	838 (4.94%)	9,848 (9.35%)	1,530 (4.71%)	4,247 (5.67%)	3,254 (3.42%)	926 (4.31%)	41 (2.48%)	279 (3.70%)	<0.001
Trichomones	303 (1.82%)	267 (1.57%)	1,360 (1.29%)	446 (1.37%)	950 (1.27%)	1,460 (1.54%)	239 (1.11%)	23 (1.39%)	99 (1.31%)	<0.001
Thrombocytopenia	557 (3.34%)	436 (2.57%)	1,755 (1.67%)	1,303 (4.01%)	1,840 (2.46%)	3,753 (3.95%)	745 (3.47%)	61 (3.69%)	244 (3.23%)	<0.001
Tuberculosis	186 (1.12%)	115 (0.68%)	622 (0.59%)	249 (0.77%)	375 (0.50%)	457 (0.48%)	151 (0.70%)	12 (0.73%)	58 (0.77%)	<0.001
Xancroid	5 (0.03%)	2 (0.01%)	21 (0.02%)	8 (0.02%)	12 (0.02%)	16 (0.02%)	8 (0.04%)	1 (0.06%)	4 (0.05%)	0.202
Kaposi's sarcoma	4 (0.02%)	0 (0.00%)	11 (0.01%)	9 (0.03%)	9 (0.01%)	10 (0.01%)	3 (0.01%)	0 (0.00%)	2 (0.03%)	0.172
Other defining	30 (0.18%)	14 (0.08%)	382 (0.36%)	89 (0.27%)	68 (0.09%)	166 (0.17%)	20 (0.09%)	7 (0.42%)	17 (0.23%)	<0.001
Other STIs	316 (1.90%)	221 (1.30%)	2,070 (1.97%)	725 (2.23%)	715 (0.96%)	2,679 (2.82%)	375 (1.74%)	16 (0.97%)	65 (0.86%)	<0.001

Absolute and relative frequencies of the different indicator conditions in the total diagnostic episodes including an indicator condition, globally, and by Sanitary Region. P-values contrast homogeneity in the distribution of the relative presence of such indicator condition between sanitary regions.
